# Supplementary material for: “I just felt supported”: Transgender and non-binary patient perspectives on receiving transition-related healthcare in family planning clinics
Source: PLoS One. 2022 Jul 21;17(7):e0271691. doi: 10.1371/journal.pone.0271691 (PMC9302788; doi:10.1371/journal.pone.0271691)
Supplement: Attachment — Submitted filename: Response to Reviewers.docx [file pone.0271691.s001.docx]

April 12, 2022,

***PLOS One***

Dear Editors and Reviewers:

Thank you for the opportunity to revise our manuscript: "’I just felt supported’: Transgender and Non-Binary Patient Perspectives on Receiving Transition-Related Healthcare in Family Planning Clinics”, which we are submitting for exclusive consideration of publication in ***PLOS One***.

Editorial changes: We have made the requested editorial changes to the manuscript format. We have removed the funding statement from the manuscript as well. The Methods section already contained the IRB name and consent information, but we have added this as a specific ethics statement section to clearly highlight this. This section includes the note that all names used in the manuscript are pseudonyms.

Please see below for a detailed response to each reviewer comment:

**Reviewer #1:**

*1) 'Transgender and non-binary' could be read as implying that non-binary people are not trans. Is there a clearer way to state that the manuscript talks about binary and non-binary people (without implying that all non-binary people refer to themselves as trans)? One way might be to use Florence Ashley's term 'gender modality'.*

Thank you for the suggestion of the Florence Ashley term. This term was new to our team. Although all participants in our study identified as transgender, including the non-binary participants, we recognize that the current language may not reflect all non-binary people’s experiences. We have changed the introduction to reflect transgender and/or non-binary people as a starting place and included a reference to the Florence Ashley pre-print piece on gender modality as suggested language moving forward in the discussion section.

*2) The introduction should more clearly state what is 'transition related care' in family planning clinics. My first thought was contraception or abortion, but these arent inherently 'transition related' (though they may be gender affirming). This is explained a little in the method, but for an international readership this should be outlined at the start of the article - it would be atypical to access hormones in family planning clinics in many countries outside the US.*

We have added information to the introduction to clearly state that in this context, we are referring to gender affirming care for medical transition, primarily hormone therapy.

*3) I personally would prefer to see extracts in the body of the text and discussed, rather than in a table. Having them solely in a table treats qualitative data as an abstracted entity.*

*4) The extracts in a table makes the results rather odd. The themes are just summaries of a rather large data set, yet despite this being very codified and reductive, no prevalence is given (ie as per a content analysis). There is a sense in which the way the results are presented doesnt do service to either the data or thematic analysis.*

Thank you for these two related comments. We agree. While the table formatting was an attempt to be more succinct, we recognize (as in comment 4) that this distracts from our methodological process and analysis. We have integrated participant quotes throughout the results section to reduce the abstraction and connect participant experiences to themes.

*5) I'm really not sure this is the 'first' study (and that language is itself colonising). A simple google scholar search for 'trans fertility planning' shows a number of studies on the first page.*

We have amended this language to reflect that ours is one of the few studies to consider the intersection of transition-related care at family planning clinics. While it is challenging to know if you are the first (and we recognize the potential colonizing language of this), our review of the literature does show that several studies exist about transgender and/or non-binary people and family planning care (contraception, abortion), there were no other studies on those patients getting their hormone therapy in this particular clinic context.

**Reviewer #2:**

Thank you for your initial comments on the study.

*1 - In line 132 the authors present: "Twenty-three (23) of the 34 were White only (67%), 7 (20%) were biracial or multiracial (non-Latinx), and the remaining 3 (9%) were Black, Middle Eastern, or East Asian only". These dice add up to 33, not 34.*

Thank you for catching this error in transcribing our participant demographics. We have updated Table 1 and the paragraph describing participants to match each other and our sample accurately.

*2 - In line 134, the data on the profile of participants show only Latinos (N=6 or 18%). Do the authors not consider it important to characterize the others? I ask this because other research suggests that access for vulnerable people, such as transgender people, for example, is strongly associated with intersections of gender, race, class, region, etc. In the data of this research, intersectionality is evidenced and recommended, in the conclusions, as an object of further studies.*

When updating the demographics, we also changed how we referenced participants who identified as Latinx (either only Latinx or as part of their biracial identity).

*3 - Line 136 - shows data on the time the person uses or interacts with the health service. Considering the objective of the article, I suggest deepening this data, considering that positive or negative experiences in health services also result from the quality of interaction and link between people involved in providing and receiving care.*

This was an interesting comment – we had not noticed any trends or differences between participants who had only been to clinics once vs. participants who had been going for longer periods of time. We did revisit our data and add in a note in the results section under positive experiences that none of the participants would decline to return for HT services, though we did not have data about if this was only due to access vs experience with treatment. We also added a piece about participants forming relationships with providers for those who had been going to their clinic for several months. We also added a note on this to our discussion section, linking positive experiences with continuation of care vs. delays in care as noted in previous literature.

*4 - Line 140 - Mention some services that people seek including "other screening services performed". My suggestion is that you can give some examples of these other experiences. This can contribute to the clarification of mistaken and reductionist ideas about the health needs of these people and practices that are limited to body transformation or sexually transmitted infections, for example*.

We have added a line with examples of screening services performed – pap smears and chest exams.

*5 - In Line 146, "Table 1: Participant Demographics (n=34)" I suggest reviewing the data: About gender, in the table an agender person is presented. My suggestion is to present this person, in the text, as part of the group of non-binary people. Also, I was in doubt about the data related to Latinos: in the text six Latin people are mentioned and in the table, two. This consideration is related to item 2 of my assessment*.

We have amended Table 1 to combine these two categories, as suggested. We also revised the text description to clarify the number of Latinx participants.

Thank you for considering this revision of our work. Please address all correspondence concerning this manuscript to:

Natalie Ingraham, PhD, MPH
Assistant Professor

CSU East Bay, Dept. of Sociology

25800 Carlos Bee Blvd, Hayward, CA 94542
E-mail: [natalie.ingraham@csueastbay.edu](mailto:natalie.ingraham@csueastbay.edu)	Phone: (510)736-3075

Sincerely,

Natalie Ingraham, PhD, MPH

Lindsey Fox, BA

Andres Leon Gonzalez, MSW

Aerin Riegelsberger, MSW
